# Microglial Kv1.3 Channels and P2Y12 Receptors Differentially Regulate Cytokine and Chemokine Release from Brain Slices of Young Adult and Aged Mice

**DOI:** 10.1371/journal.pone.0128463

**Published:** 2015-05-26

**Authors:** Nicoletta Charolidi, Tom Schilling, Claudia Eder

**Affiliations:** Institute for Infection and Immunity; St. George’s, University of London; Cranmer Terrace; London SW17 0RE, United Kingdom; Indiana School of Medicine, UNITED STATES

## Abstract

Brain tissue damage following stroke or traumatic brain injury is accompanied by neuroinflammatory processes, while microglia play a central role in causing and regulating neuroinflammation via production of proinflammatory substances, including cytokines and chemokines. Here, we used brain slices, an established *in situ* brain injury model, from young adult and aged mice to investigate cytokine and chemokine production with particular focus on the role of microglia. Twenty four hours after slice preparation, higher concentrations of proinflammatory cytokines, i.e. TNF-α and IL-6, and chemokines, i.e. CCL2 and CXCL1, were released from brain slices of aged mice than from slices of young adult mice. However, maximal microglial stimulation with LPS for 24 h did not reveal age-dependent differences in the amounts of released cytokines and chemokines. Mechanisms underlying microglial cytokine and chemokine production appear to be similar in young adult and aged mice. Inhibition of microglial Kv1.3 channels with margatoxin reduced release of IL-6, but not release of CCL2 and CXCL1. In contrast, blockade of microglial P2Y12 receptors with PSB0739 inhibited release of CCL2 and CXCL1, whereas release of IL-6 remained unaffected. Cytokine and chemokine production was not reduced by inhibitors of Kir2.1 K+ channels or adenosine receptors. In summary, our data suggest that brain tissue damage-induced production of cytokines and chemokines is age-dependent, and differentially regulated by microglial Kv1.3 channels and P2Y12 receptors.

## Introduction

Stroke and traumatic brain injury cause substantial tissue damage and subsequent neuroinflammation. Neuroinflammatory processes can have beneficial and detrimental effects and are mainly driven by microglial cells via production of proinflammatory cytokines and chemokines [1–6]. To date, it is not fully understood by which mechanisms microglial cytokine and chemokine production is triggered and maintained following brain damage. It has been shown that in the injured brain, ATP is rapidly released from damaged cells [5, 7] and triggers microglial process extension towards brain lesions via stimulation of P2Y12 receptors [8]. However, within a few hours after microglial activation, P2Y12 receptors are downregulated, while A_2A_ adenosine receptors are upregulated simultaneously. Adenosine receptor stimulation mediates subsequent microglial process retraction, resulting in complete transformation of microglia from their ramified into ameboid morphology in less than 24 hours [9]. In addition to ATP, UDP is released following neuronal damage leading to enhanced microglial phagocytosis and chemokine expression via stimulation of microglial P2Y6 receptors [10, 11]. Furthermore, upregulation of K^+^ channels is a hallmark of microglial activation. Enhanced expression of inward rectifier Kir2.1 and outward rectifier Kv1.3 K^+^ channels has been demonstrated in activated microglia *in vitro* [12], *in situ* [13] and *in vivo* [14–17]. To date, it remains unclear whether activation of K^+^ channels and/or stimulation of P2Y12 or adenosine receptors are required for microglial cytokine and chemokine production following brain tissue damage.

Intriguingly, expression of Kir2.1 and Kv1.3 K^+^ channels as well as of P2Y12 receptors is also enhanced in microglial cells of aged mice compared to young adult mice [18–20]. High expression of Kv1.3 channels has also been found in microglia of patients with Alzheimer’s disease [17]. In addition to ATP receptor and K^+^ channel upregulation, aging causes a variety of changes in microglial properties and behavior. Microglial cells in the aged brain are characterized by dystrophic morphology, reduced motility and enhanced production of proinflammatory cytokines and chemokines, among others. It is still a matter of debate whether microglia in the aged brain are shifted towards a primed, proinflammatory state or become less capable of performing their normal functions [21–25].

In this study, we investigated cytokine and chemokine release from brain slices of young adult and aged mice to gain a better understanding of early neuroinflammatory processes occurring rapidly following damage of young and aged brain tissue. Furthermore, we aimed to identify microglial purinergic receptors and K^+^ channels involved in the release of proinflammatory cytokines and chemokines.

## Materials and Methods

In accordance with the United Kingdom Animal (Scientific Procedures) Act of 1986, this study did not require a Home Office project license because no regulated procedures were carried out. Mice were humanely killed at a designated establishment by dislocation of the neck, which is an appropriate method under Schedule 1 of the Act.

### Preparation and maintenance of brain slices

Coronal brain slices were prepared from young adult (2–3 months, 17 animals in total) and aged (21–24 months, 16 animals in total) female C57BL6 mice (Harlan Laboratories, Bicester, UK) as described previously [20]. In brief, after dislocation of the neck, mice were decapitated and the brain was removed. Tissue blocks of the frontoparietal lobes were mounted on a vibratome (Dosaka, Kyoto, Japan) in a chamber filled with gassed (95% O_2_, 5% CO_2_) ice-cold HEPES-containing preparation medium (MEM, pH 7.35; Life Technologies, Paisley, UK) and slices of 300 μm thickness were made under sterile conditions. On average, 12 slices per brain were prepared. Each freshly prepared brain slice was placed on a Millicell culture plate insert (12 μm pore size; Merck Millipore, Darmstadt, Germany) and transferred into 24-well plates containing 800 μl serum-free medium (DMEM, pH 7.4; Life Technologies, Paisley, UK) with or without LPS or channel/receptor inhibitors. In experiments using ML133, the medium of control slices contained additionally 0.1% DMSO. After this initial incubation for 30 min, the medium above slices was removed and slices were kept for 24 hours in 350 μl serum-free DMEM with or without LPS or channel/receptor inhibitors as indicated.

### Chemicals

The following drugs were used in this study: 100 μM caffeine; 1 μg/ml lipopolysaccharide (LPS); 1 μM margatoxin (MTX; PeptaNova, Sandhausen, Germany); 20 μM N-[(4-Methoxyphenyl)methyl]-1-naphthalenemethanamine hydrochloride (ML133; R&D Systems, Abingdon, UK); 10 μM (1-amino-4-[4-phenylamino-3-sulfophenylamino]-9,10-dioxo-9,10-dihydroanthracene-2-sulfonate (PSB0739; R&D Systems, Abingdon, UK). A stock solution of 20 mM ML133 was prepared in DMSO. All other drugs were prepared in DMEM (Life Technologies, Paisley, UK). If not stated otherwise drugs/chemicals were obtained from Sigma-Aldrich, Dorset, UK.

### Detection of cytokines and chemokines by ELISA

After incubation of brain slices in a cell culture incubator at 37°C for 24 h, the medium was collected and amounts of cytokines and chemokines released into the medium were determined by ELISA. Data were obtained from at least 2 individual brain slices of a minimum of 3 individual mouse preparations per experimental condition. For quantitative determination of cytokine and chemokine concentrations, Quantikine mouse TNF-α, IL-6, IL-10, CCL2, CXCL1, TGF-β and IGF-1 Immunoassay kits (all from R&D Systems, Abingdon, UK) were used according to the manufacturer's instructions.

### Normalization of cytokine and chemokine concentrations

For comparison of data between brain slices of different experimental conditions, amounts of cytokines and chemokines released into the medium were normalized to the size of brain slices. Immediately after collection of the media for subsequent ELISA analyses, pictures of slices were taken using a Canon EOS 450D camera (Canon Inc., Chichibu, Japan), and the slice surface area was determined using the program ImageJ (NIH, Bethesda, MA, USA).

### Statistics

All data are presented as mean±SEM. Statistical significance of differences between experimental groups was evaluated using the SPSS program (IBM, Armonk, NY, USA). One-way ANOVA with Dunnett’s T3 post hoc tests were used to compare cytokine release from brain slices of young adult and aged mice in presence or absence of LPS stimulation. Paired t-tests were used for comparison of the effects of receptor/channel inhibitors. For each inhibitor tested, a pair of consecutive brain slices was kept with or without inhibitor, while n represents the number of slice pairs investigated. Independent t-tests were used for comparison of cytokine/chemokine release from young adult and aged mouse slices and for comparison of brain slice area of young adult and aged mice. Equality of variances for one-way ANOVA and independent t-test calculations was assessed with Levene’s test. Data were considered to be statistically significant with p<0.05.

## Results

### Cytokine and chemokine release from brain slices of young adult and aged mice

In all experiments, brain slices were placed on culture plate inserts as described previously [13, 26], and concentrations of cytokines and chemokines released into the medium were determined. This method has previously been established to study cytokine and chemokine release from brain slices (e.g., [27, 28]), and has been shown to be optimal for keeping brain slices without causing substantial cell death/damage after the initially occurring tissue damage at the slice surface due to brain slicing [26]. In contrast to commonly used slice culture media [13, 26], serum was not added to the medium in our experiments to avoid additional activation of glial cells by serum components.

**Fig 1:**
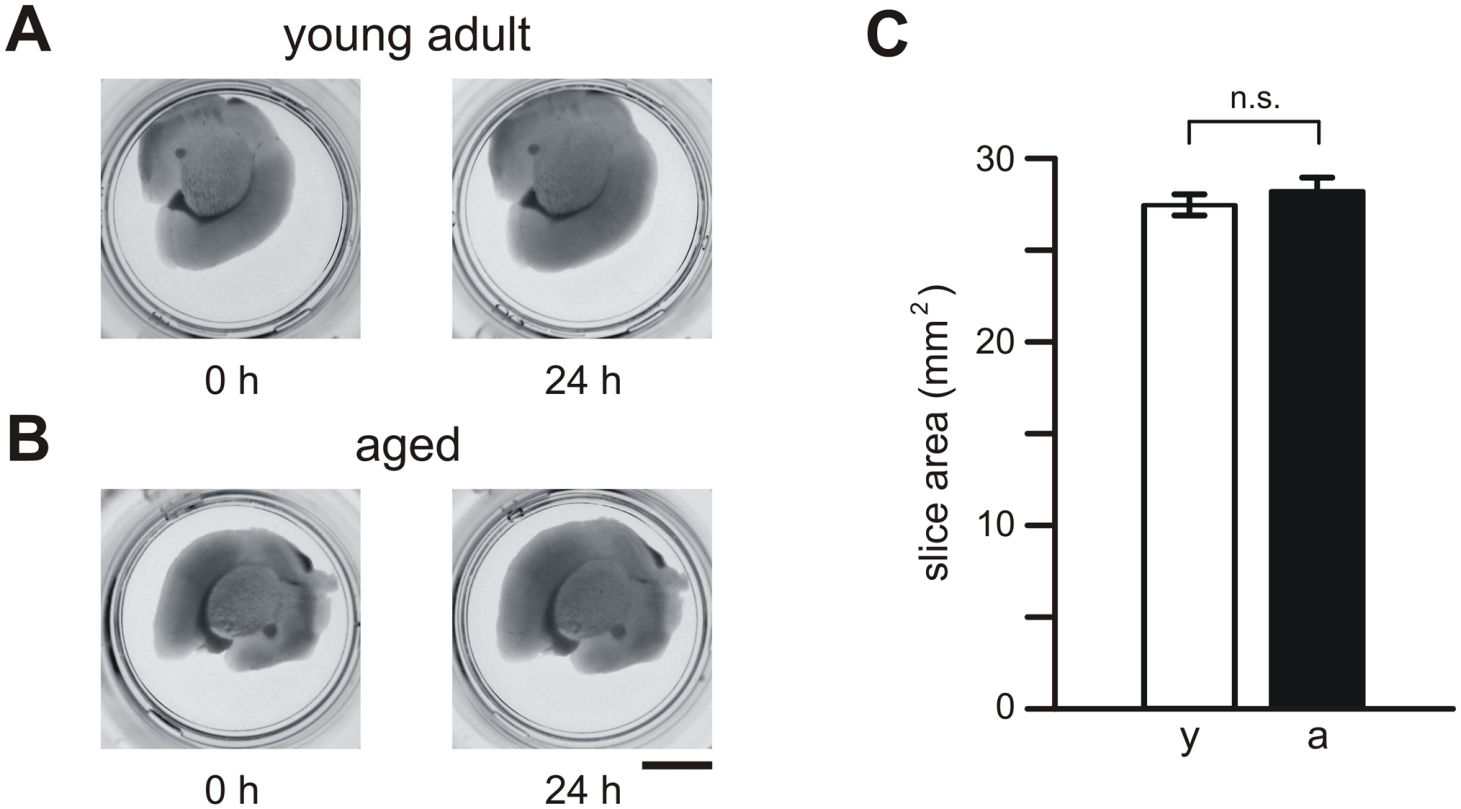
Brain slices from young adult and aged mice. (A) Pictures of individual brain slices from young adult (A) and aged (B) mice. Slices placed onto culture inserts in 24-well plates are shown immediately (0 h) and 1 day (24 h) after slice preparation. (C) Mean values (±SEM) of brain slice area determined for young adult mice (y, open bars) and aged mice (a, closed bars). Calibration bar: 5 mm; n.s., not significantly different.

Within 24 h after preparation, brain slices did not change substantially in shape or size. Photographs of brain slices taken immediately and 24 h after slice preparation are shown in Fig 1. Furthermore, slices prepared from young adult (Fig 1A) and aged (Fig 1B) mice did not differ significantly in size (p = 0.215; Fig 1C). We determined a mean area of 27.4±0.4 mm^2^ (n = 194) for brain slices of young adult mice and of 28.2±0.5 mm^2^ (n = 176) for slices of aged mice (Fig 1C).

**Fig 2:**
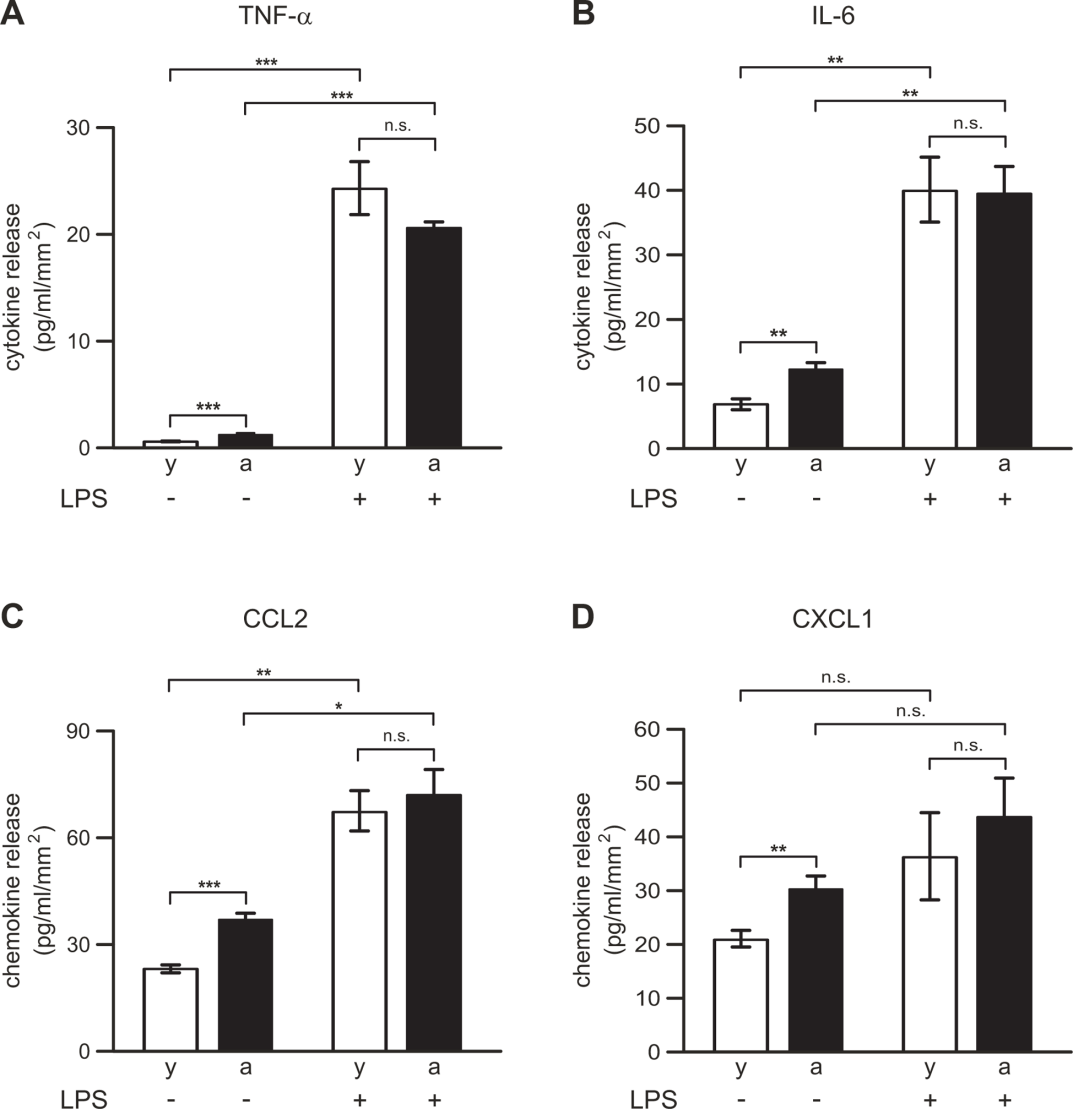
Amounts of proinflammatory cytokines and chemokines released from brain slices of young adult (y, open bars) and aged (a, closed bars) mice. (A-D) Concentrations of TNF-α (A), IL-6 (B), CCL2 (C) and CXCL1 (D) were determined in media of slices, which were either kept untreated or treated with 1 μg/ml LPS for 24 h. Cytokine and chemokine concentrations determined for individual slices were normalized to the corresponding slice area. ***, p<0.001; **, p<0.01; *, p<0.05; n.s., not significantly different.

Amounts of cytokines and chemokines released into DMEM within 24 h after slice preparations are summarized in Fig 2. Surprisingly, very small concentrations of TNF-α were detected. On average, 0.43±0.03 pg/ml/mm^2^ (n = 65) and 1.0±0.05 pg/ml/mm^2^ (n = 64) TNF-α were released from brain slices of young adult and aged mice, respectively. Comparison of TNF-α concentrations released from young adult and aged mice revealed significant differences (p<0.001), i.e. TNF-α production was 2.3-fold larger in aged mice than in young adult mice (Fig 2A). Additional stimulation of slices with 1 μg/ml LPS for 24 h induced substantial TNF-α production (Fig 2A). In comparison with untreated brain slices, LPS increased the production of TNF-α 56-fold (n = 6; p<0.001) in slices from young adult mice and 21-fold (n = 6; p<0.001) in slices from aged mice (Fig 2A). Mean concentrations of TNF-α released from LPS-stimulated brain slices of young adult and aged mice did not differ significantly (p = 0.667).

In contrast to TNF-α, substantial amounts of the proinflammatory cytokine IL-6 were released from brain slices of both young adult (6.9±0.9 pg/ml/mm^2^, n = 38) and aged (12.4±0.8 pg/ml/mm^2^, n = 61) mice (Fig 2B). On average, concentrations of IL-6 released from slices of aged mice were 1.8-fold higher (p<0.01) than those released from slices of young adult mice. Stimulation with 1 μg/ml LPS for 24 h further enhanced production of IL-6 in brain slices. LPS-treated slices released 5.8-fold (p<0.01, n = 8) and 3.2-fold (p<0.01, n = 6) higher concentrations of IL-6 than untreated slices of young adult and aged mice, respectively (Fig 2B). Comparison of mean concentrations of IL-6 released from LPS-treated slices of young adult and aged mice did not reveal significant differences (p = 1.0).

The chemokines CCL2 (23.1±1.0 pg/ml/mm^2^, n = 96 for young adult mice; 37.2±1.5 pg/ml/mm^2^, n = 86 for aged mice) and CXCL1 (21.1±1.5 pg/ml/mm^2^, n = 97 for young adult mice; 30.4±2.3 pg/ml/mm^2^, n = 86 for aged mice) were also released at high concentrations (Fig 2C and 2D). Similar to observations made for cytokine production (see above), in the absence of LPS, chemokine production was found to be age-dependent. In comparison with data obtained from young adult mice, 1.6-fold more CCL2 (p<0.001) and 1.4-fold more CXCL1 (p<0.01) were released from slices of aged mice (Fig 2C and 2D). Stimulation of brain slices with LPS caused further increases in chemokine production (Fig 2C and 2D). In the presence of LPS, CCL2 production was increased 2.9-fold (p<0.01, n = 6) and 1.9-fold (p<0.05, n = 6) in young adult and aged mouse slices, respectively. However, no significant differences were found in comparison of mean CCL2 concentrations released from LPS-stimulated slices of young adult and aged mice (p = 0.993). In the presence of LPS, CXCL1 production was slightly, but not significantly, increased in slices of young adult (1.7-fold increase, p = 0.435, n = 6) and aged (1.4-fold increase, p = 0.459, n = 6) mice compared to the corresponding slices kept in the absence of LPS. The LPS-induced release of CXCL1 did not differ significantly between young adult and aged mice (p = 0.977).

Furthermore, very small, if any, concentrations of the anti-inflammatory substances TGF-β (<0.1 pg/ml/mm^2^ (n = 71) for young adult mice; <0.9 pg/ml/mm^2^ (n = 70) for aged mice), IL-10 (<0.1 pg/ml/mm^2^ (n = 71) for young adult mice; <0.1 pg/ml/mm^2^ (n = 70) for aged mice) and IGF-1 (<0.5 pg/ml/mm^2^ (n = 71) for young adult mice; <0.5 pg/ml/mm^2^ (n = 70) for aged mice) were detected in media collected from brain slices of young adult and aged mice 24 h after slice preparation.

### Role of microglial P2Y12 receptors and Kv1.3 channels in cytokine and chemokine release

Next, we aimed to identify mechanisms by which microglial production of proinflammatory cytokines and chemokines is regulated in young adult and aged mice. For pharmacological characterization, we investigated exclusively release of IL-6, CCL2 and CXCL1, which were produced at high concentrations.

**Fig 3:**
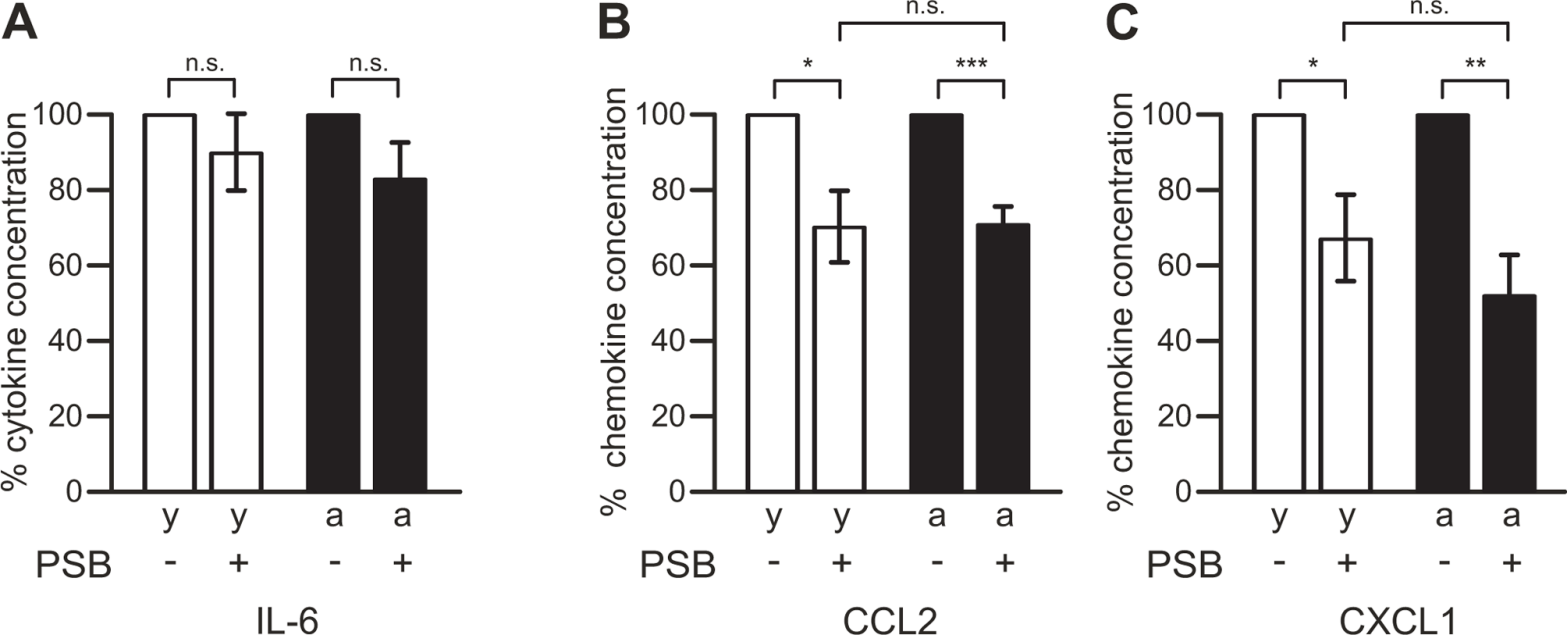
Role of microglial P2Y12 receptors in chemokine, but not cytokine, release. Concentrations of IL-6 (A), CCL2 (B) and CXCL1 (C) were determined for young adult (y, open bars) and aged (a, closed bars) mice. Cytokine/chemokine concentrations determined in media from slices treated with 10 μM PSB0739 (PSB) were normalized to those from control slices kept untreated for 24 h. ***, p<0.001; **, p<0.01; *, p<0.05; n.s., not significantly different.

First, we investigated whether cytokine and chemokine release from brain slices is mediated via microglial P2Y12 receptor stimulation. As demonstrated in Fig 3, inhibition of microglial P2Y12 receptors with 10 μM PSB0739 did not significantly inhibit release of the proinflammatory cytokine IL-6 from slices of young adult (p = 0.351, n = 9) and aged (p = 0.116, n = 10) mice (Fig 3A). However, PSB0739 significantly reduced the amounts of released chemokines. In the presence of 10 μM PSB0739, release of CCL2 was inhibited by 30±9% (n = 9, p<0.05) in slices from young adult mice, and by 29±5% (n = 10, p<0.001) in slices from aged mice (Fig 3B). Similarly, CXCL1 release was affected by P2Y12 receptor inhibition with PSB0739 (Fig 3C). In comparison with untreated controls, the mean concentration of CXCL1 determined in the medium from PSB0739-treated slices of young adult mice was reduced by 33±11% (n = 10, p<0.05), while CXCL1 production was inhibited by 48±11% (n = 10, p<0.01) in PSB0739-treated slices from aged mice. Statistical analyses of the PSB0739-induced percentage inhibition of chemokine release from brain slices of young adult and aged mice did not reveal significant differences between both age groups (p = 0.971 for CCL2; p = 0.335 for CXCL1).

**Fig 4:**
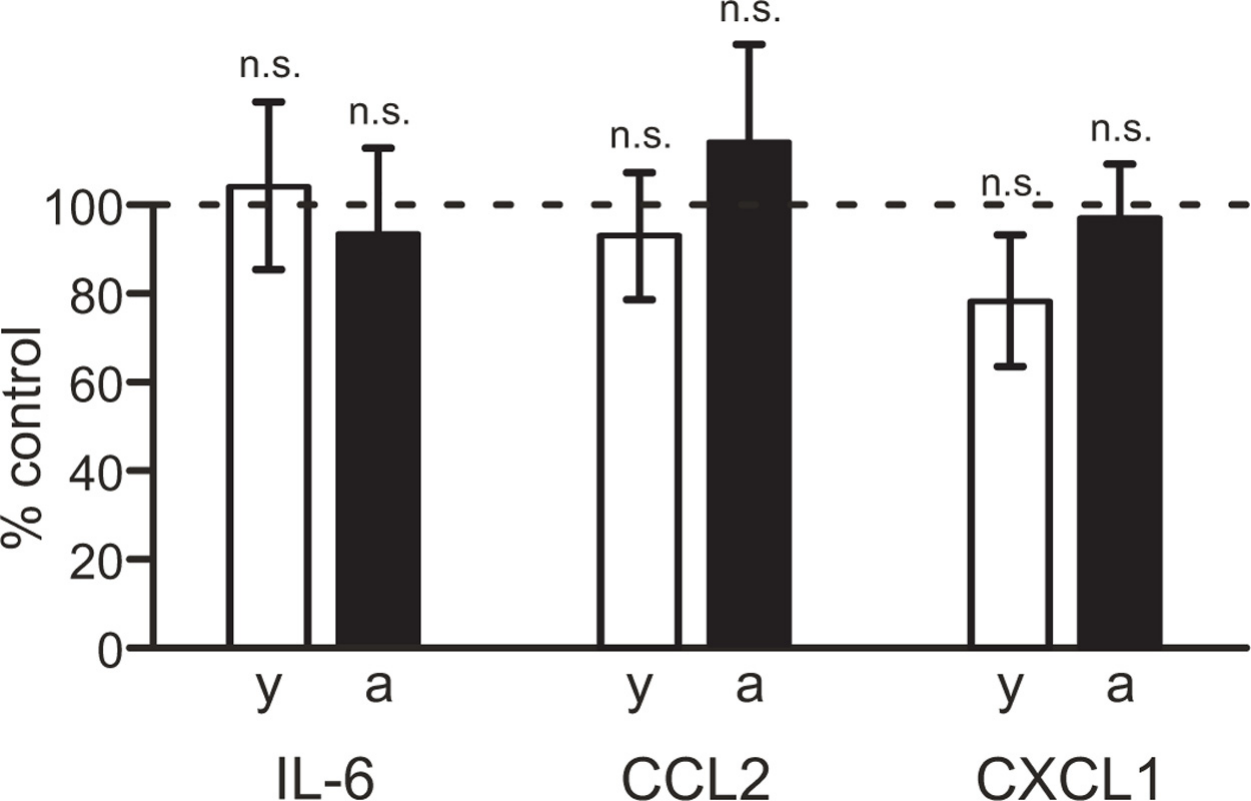
Lack of involvement of adenosine receptors in regulating cytokine and chemokine production in brain slices. Release of IL-6, CCL2 and CXCL1 from brain slices of young adult (y, open bars) and aged (a, closed bars) mice was determined in absence or presence of 100 μM caffeine. Cytokine/chemokine concentrations determined in media from caffeine-treated slices are presented as percentage of cytokine/chemokine concentrations determined in media from corresponding untreated control slices. n.s., not significantly different.

In order to identify a possible involvement of adenosine receptors in the regulation of cytokine/chemokine release from brain slices, we tested effects of the broad-spectrum adenosine receptor inhibitor caffeine. As demonstrated in Fig 4, inhibition of adenosine receptors with 100 μM caffeine did not significantly affect the release of IL-6, CCL2 or CXCL1 from brain slices of either young adult or aged mice (young adult mice: p = 0.785, n = 10 for IL-6, p = 0.694, n = 10 for CCL2, p = 0.210, n = 10 for CXCL1; aged mice: p = 0.783, n = 10 for IL-6, p = 0.496, n = 10 for CCL2, p = 0.903, n = 10 for CXCL1).

**Fig 5:**
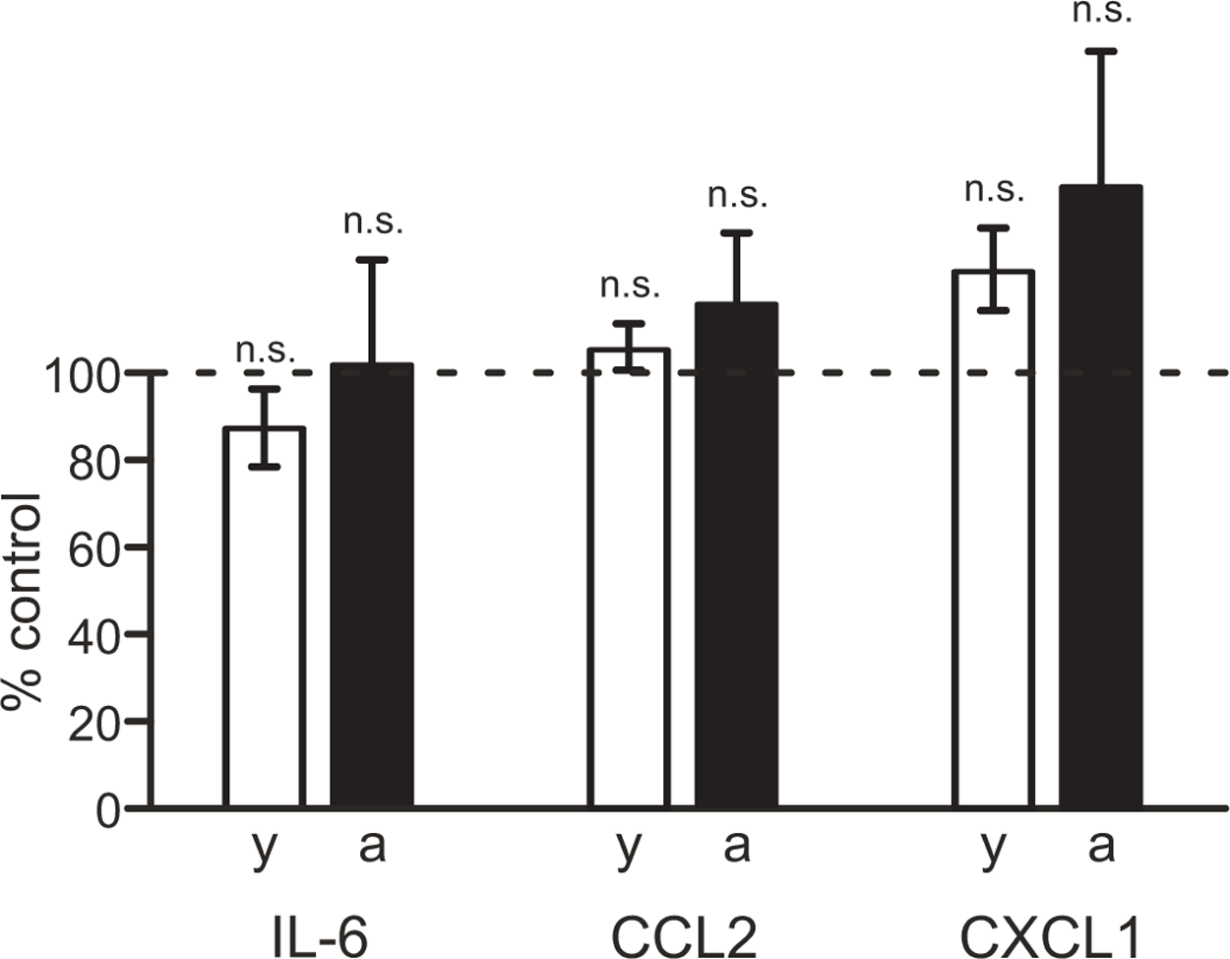
Lack of involvement of microglial Kir2.1 K^+^ channels in regulating cytokine and chemokine release from brain slices. Release of IL-6, CCL2 and CXCL1 from brain slices of young adult (y, open bars) and aged (a, closed bars) mice was determined in absence or presence of 20 μM ML133. Cytokine/chemokine concentrations determined in media from ML133-treated slices are presented as percentage of cytokine/chemokine concentrations determined in media from corresponding untreated control slices. n.s., not significantly different.

Next, we determined the role of Kir2.1 inward rectifier K^+^ channels and Kv1.3 outward rectifier K^+^ channels. To test whether microglial cytokine and/or chemokine production in young adult and aged mouse slices is regulated by inward rectifier K^+^ channel activity, Kir2.1 channels were blocked with 20 μM ML133, a concentration 10-times higher than the IC50 value for ML133-induced channel inhibition and sufficient to induce complete channel blockade [29]. As demonstrated in Fig 5, ML133 did not inhibit cytokine or chemokine release from brain slices of young adult and aged mice. No significant differences were found in the amounts of IL-6, CCL2 and CXCL1 released from brain slices kept untreated or treated with 20 μM ML133 (young adult mice: p = 0.227, n = 5 for IL-6, p = 0.355, n = 5 for CCL2, p = 0.068, n = 5 for CXCL1; aged mice: p = 0.935, n = 7 for IL-6, p = 0.354, n = 7 for CCL2, p = 0.211, n = 7 for CXCL1).

**Fig 6:**
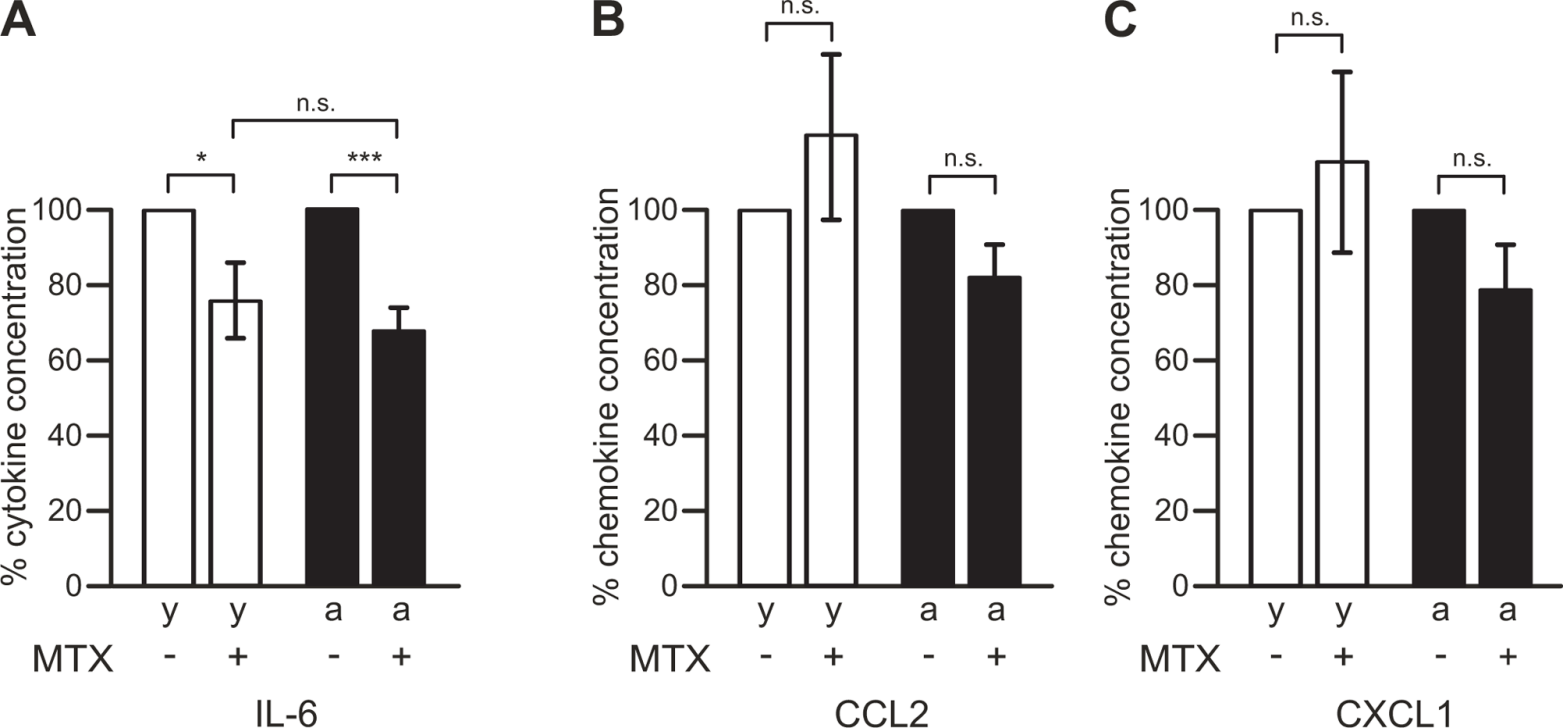
Role of microglial Kv1.3 K^+^ channels in cytokine, but not chemokine, release. Concentrations of IL-6 (A), CCL2 (B) and CXCL1 (C) were determined for young adult (y, open bars) and aged (a, closed bars) mice. Cytokine/chemokine concentrations determined in media from slices treated with 1 μM MTX were normalized to those determined in media from control slices kept untreated for 24 h. ***, p<0.001; *, p<0.05; n.s., not significantly different.

In contrast, inhibition of Kv1.3 channels with 1 μM MTX significantly reduced the production of IL-6 by 24±10% (n = 10; p<0.05) in brain slices from young adult mice, and by 32±6% (n = 8; p<0.001) in brain slices from aged mice, respectively (Fig 6A). The MTX-induced percentage inhibition of IL-6 production in slices of young adult mice was not significantly different (p = 0.538) from that determined for aged mouse slices. MTX inhibited exclusively the release of IL-6 from brain slices. Release of CCL2 (p = 0.384, n = 10 for young adult mice; p = 0.085, n = 8 for aged mice) and of CXCL1 (p = 0.606, n = 10 for young adult mice; p = 0.115, n = 8 for aged mice) remained unaffected by Kv1.3 channel inhibition with MTX (Fig 6B and 6C).

## Discussion

In this study, we investigated production of cytokines and chemokines immediately after brain tissue slicing. Using this *in situ* model, we mimicked early neuroinflammatory processes occurring rapidly following acute brain tissue damage. Freshly prepared and/or organotypic brain slices have frequently been used in the past in order to study microglial activation in brain tissue [8, 9, 27, 30], and have been suggested for investigations of microglial behavior rather than isolated, cultured microglia [31]. Recent publications have questioned the reliability of data obtained from isolated/cultured microglial cells, as cell culture conditions do not fully resemble the environment of microglial cells within the brain [31]. Another advantage of using brain slices is the possibility to study exclusively microglial responses, which often is impossible *in vivo* due to infiltration of peripheral monocytes/macrophages into the injured brain following disruption of the blood-brain barrier. However, this model also has some disadvantages, as different cell types contribute to the release of cytokines and chemokines and might be affected by channel/receptor inhibitors. For example, in addition to microglia, astrocytes have been found to produce IL-6 following brain damage [32], and some neurons have been identified to express Kv1.3 channels [33].

In summary, we found that tissue damage due to slicing of the brain caused rapid increases in the production of proinflammatory, but not anti-inflammatory, cytokines and chemokines. In agreement with previously obtained *in situ* and *in vivo* data from damaged brain tissue [27, 34], high concentrations of the proinflammatory cytokine IL-6 and of the chemokines CCL2 and CXCL1 were detected 24 h after slice preparation. Surprisingly, release of the proinflammatory cytokine TNF-α was very small in the absence of LPS stimulation, whereas large amounts of TNF-α were released from LPS-treated slices within the same time course. These data suggest that damage-associated molecular pattern molecules (DAMPs), i.e. molecules released from damaged cells, such as ATP, and pathogen-associated molecular pattern molecules (PAMPs), such as LPS, stimulate different pathways leading to the release of different cytokines and chemokines in brain tissue. This observation is in agreement with previous findings that DAMPs and PAMPs trigger the assembly of different inflammasomes and modulate downstream distinct intracellular signaling pathways in microglia and other macrophages [35].

Results of our study demonstrate that damage-associated cytokine and chemokine production is age-dependent, i.e., higher cytokine and chemokine concentrations were determined in media from aged mouse slices than in media from young mouse slices. This finding can be explained by different mechanisms: One possibility is that neuronal death induced by the preparation of brain slices is more pronounced in aged than in young adult mouse slices, leading to more pronounced glial activation and subsequently to enhanced cytokine/chemokine release from slices of aged mice. Another possibility is that aged microglial cells are in a primed state, and, therefore, produce more cytokines and chemokines upon stimulation. Priming of microglia in the aged brain has been previously reported [23]; it may include a lower activation threshold, higher sensitivity or enhanced expression of receptors, stronger phosphorylation of certain intracellular components, more efficient release mechanisms, among others. In agreement with this hypothesis and our observation, it has been reported that unstimulated isolated microglial cells from aged mice were characterized by upregulated mRNA levels of proinflammatory cytokines and chemokines and released higher concentrations of proinflammatory cytokines than isolated microglia from young mice [36, 37]. Intriguingly, mimicking pathogen-stimulated cytokine and chemokine production by treating slices with LPS did not reveal age-dependent differences in cytokine and chemokine production. It is possible that the total capacity of microglia to produce cytokines and chemokines upon maximal stimulation with LPS is independent of the cells’ priming state.

Although several cell types produce cytokines and chemokines following brain injury, in this study, we focused exclusively on identifying mechanisms underlying microglial cytokine and chemokine production. We did not observe significant differences between young adult and aged mice in the percentage inhibition of cytokine or chemokine production by inhibitors of microglial purinergic receptors or K^+^ channels. These data suggest that aging does not cause substantial changes in mechanisms by which microglial cytokine and chemokine production is regulated. In summary, we have found that microglial P2Y12 receptors and Kv1.3 channels differentially regulate cytokine and chemokine release after brain tissue damage. It has been demonstrated that ATP released from damaged cells following laser ablation in brain slices rapidly activates microglia via P2Y12 receptor stimulation, causing extension of microglial processes towards the brain lesion [8]. Furthermore, P2Y12 receptor-mediated process extension towards hyperactive neurons has recently been reported [38, 39]. Here we demonstrate an additional function of microglial P2Y12 receptors, namely regulation of the release of the chemokines CCL2 and CXCL1. In brain pathology, CCL2 mainly recruits local microglia as well as peripheral monocytes and macrophages, whereas neutrophils are recruited by CXCL1 [5, 40]. Thus, inhibition of microglial P2Y12 receptors following stroke or brain injury could reduce the amount of infiltrating leukocytes, which subsequently would promote brain repair mechanisms and improve disease recovery.

Inhibition of microglial Kv1.3 channels has an opposite effect to blocking microglial P2Y12 receptors, namely MTX inhibited release of the proinflammatory cytokine IL-6 without affecting release of the chemokines CCL2 and CXCL1. However, we cannot completely rule out that MTX may have caused additional effects on neuronal Kv1.2 channels [41]. Further experiments are required to identify the precise mechanism by which microglial Kv1.3 channels regulate the release of IL-6. We have previously identified K^+^ channels in regulating the secretion of IL-1β via induction of a negative membrane potential [42]. It is possible that membrane hyperpolarization mediated by Kv1.3 channel activity is required for optimal IL-6 production or release, e.g. to maintain a large driving force for Ca^2+^ entry [43].
